# From Electrochemical Biosensors to Biomimetic Sensors Based on Molecularly Imprinted Polymers in Environmental Determination of Heavy Metals

**DOI:** 10.3389/fchem.2017.00047

**Published:** 2017-07-06

**Authors:** Cosimino Malitesta, Sabrina Di Masi, Elisabetta Mazzotta

**Affiliations:** Laboratorio di Chimica Analitica, Dipartimento di Scienze e Tecnologie Biologiche ed Ambientali, Università del SalentoLecce, Italy

**Keywords:** electrochemical sensors, heavy metals, biosensors, biomimetic sensors, imprinted polymers, electrosynthesized polymers

## Abstract

Recent work relevant to heavy metal determination by inhibition-enzyme electrochemical biosensors and by selected biomimetic sensors based on molecularly imprinted polymers has been reviewed. General features and peculiar aspects have been evidenced. The replace of biological component by artificial receptors promises higher selectivity and stability, while biosensors keep their capability of producing an integrated response directly related to biological toxicity of the samples.

## Introduction

Heavy metals represent a serious risk for health and environment, mainly by disruption of metabolic functions, along with their accumulation and low clearance rate. Their determination is particularly challenging due to the need of application also at very low concentrations where chronic toxicity is still observed and to toxicity relation with element speciation.

Spectroscopic and chromatographic techniques have been developed for this task and hyphenated techniques have been proposed for the most complex problems. In general, these techniques are costly, time-spending and require expert personal. In addition, is generally impossible to apply them on field.

Electroanalytical techniques are attractive alternatives (in respect to the above limits) and often they can be performed with miniaturized devices suitable for on-field measurements. In this case, selectivity, a major requirements of any analytical method for heavy metals, is mainly assured by control of potential of the working electrode. Even so, this is not adequate for resolving some interferences in voltammetric techniques (see e.g., the case of Pb and Sn which have close standard potentials) and represents a pivotal problem in amperometric and potentiometric techniques. The general solution to this drawback is to employ an electrode surface selective *per se* so to allow in principle detection only of the species of interest.

Electrochemical sensors are such a type of device. They consist, as in general chemical sensors, of a recognition element bounded to surface of the electrode (of the surface transducer in general), whose task is to interact specifically (or at least selectively) with the target analyte. As a result of interaction, a chemical change occurs at the transducer which transforms it in an electrical signal. In the case of an electrode, the signal is often an electrolysis current produced by some chemical species involved in the recognition process or the equilibrium potential established at the same electrode by the cited chemical species. The presence of a recognition element, incorporates chemical knowledge, which is otherwise external to device in classical approaches (in the software of interpretation of measurements and in the education of the operator). This reduces expertise requirements of people operating the devices.

To fulfill selectivity requests of chemical sensors researchers have considered what Nature has done in this respect: some biological objects have specifically evolved to perform specialized tasks in living organisms. Enzymes, antibodies, nucleic acids, receptors, etc. are biological components with such characteristics and have been employed as recognition elements since the seminal work of Clark and Lyons (1962) in sensors called, as a matter of fact, biosensors.

Since then a plethora of biosensors have been developed in research and some of them have reached the market and are all over the world used (see e.g., glucose biosensors employed by diabetic persons).

Applying the same concept to heavy metal determination is not straightforward as there are not many natural biological components specifically developed by living organisms for those targets. Some of them are xenobiotic and toxic also at very low concentrations and defense mechanisms involve production of proteins sequestering them. Others are essential parts of biological components (e.g., enzymes) and are toxic at higher concentrations. In all the above cases, the application to biosensing of those biological components is not so easy.

The most common approach appears to apply their toxicity mechanisms, as that represented by inhibition of enzymatic activity (see e.g., Malitesta and Guascito, 2005; Guascito et al., 2008, 2009; Malitesta et al., 2012), in detection principle. In this framework, kinetics of inhibition is an important factor in selecting enzymes whose activity must be influenced by heavy metals. In particular, reversible inhibition is the preferred tool for developing biosensors for heavy metals, even if also irreversible ones can be considered in disposable biosensors.

This kind of biosensors for heavy metals are generally not highly selective neither in respect to different elements nor in respect to different species of the same element. This is not surprising as heavy metals are not the targets for which biological components have been designed and *per se* inhibition mechanisms are less specific than recognition ones. On the other hand, they have two advantages: (1) they can be used in screening samples whose detailed heavy metal composition must be determined by more selective techniques, (2) their responses can be somewhat directly correlated to toxicity of the samples as they use the same biological components influenced by heavy metals in living organisms.

Nonetheless, even these biosensors suffer of the general limitations of these devices, e.g., they are fragile (with short lifetime), being based on biological components lying in a media (e.g., artificial polymers) different from the natural ones, cannot be used in harsh environments and are expensive.

To overcome these drawbacks research is active in the field of development of artificial receptors, mimicking the activity of biological components with the goal to produce biomimetic sensors. Among them, molecularly imprinted polymers (MIPs) have become of large application as they are very stable, cost-effective materials, applicable also to aggressive matrices and, in principle, they can be prepared for any target (for a review see e.g., Malitesta et al., 2012; Mazzotta et al., 2015). They consist of polymers prepared in the presence of the analyte (called template). This procedure, after washing out analyte molecules, leaves imprinted three-dimensional molecular memory in the material, which is then able to selectively recognize the same analyte in samples. In addition, MIPs can be developed, in principle, for single element or for a single species of an element, offering another advantage in respect to biological components. Recently the general field of MIP sensors enriched of coupling of MIP technology with nanotechnology. In fact, nano MIPs and/or nanomaterials combined with MIPs have been developed for improving sensor performances chiefly by the larger surface area available, the better analyte transport in MIP and/or the electrocatalytic properties of nanomaterials (Zaidi, 2017).

Both fields of biosensors and biomimetic sensors for heavy metal determination have been reviewed in reviews of wider topics (see e.g., Upadhyay and Verma, 2013 and Amine et al., 2016 for biosensors and Hande et al., 2015 for biomimetic sensors). Also the specific area of sensor based on nanomaterials has been very recently reviewed (Cui et al., 2015; Liu et al., 2017). This paper, after reviewing recent results in biosensors for heavy metals, aims to stress peculiar achievements in biomimetic sensors for heavy metals based on MIPs. In particular, our interest will be limited to electrochemically synthesized MIPs which represents an important class of MIPs in this field.

## Biosensors

Recent work relevant to electrochemical biosensors for heavy metal determination is summarized in Table 1. In general, only few enzyme systems (HRP, GOx, β-galactosidase) have been applied. Enzyme immobilization is performed by classical techniques (see e.g., Moyo et al., 2014; Ayenimo and Adeloju, 2015; Rust et al., 2015; Fourou et al., 2016) or by entrapment in electrosynthesized polymers (polypyrroles, polyanilines, etc.). In all but one case, proposed biosensor are reported as sensitive to different heavy metals and in several cases they suffer interference from several other ones, so that total heavy metal concentration represents the most reasonable result of the application of these sensors to real samples. LODs in the order of 10 nanomolar are often achieved and picomolar in one case. Several sensors exhibit reversible inhibition so that they can be quickly regenerated; this feature is generally coupled to long lifetime. Most work has considered application to real samples.

**Table 1:** A summary of analytical characteristics of biosensors for determination of heavy metal ions based on enzyme inhibition.

**Sensor**	**Metal Ions**	**Methods**	**Detection limit (M)^1^**	**Linear range (M)^1^**	**Tested interference^2^**	**Regeneration method**	**Stability^3^**	**Recovery in real samples**	**References**
MT-MWCNT/HRP^c^	Pb^2+^	Amperometry	7.55 × 10^−9^	2.78 × 10^−7^ – 1.66 × 10^−6^	Ca^2+^, Mg^2+^, Na^+^, K^+^	N.R.	SL: 10	96–104%^4^	Moyo et al., 2014
	Cu^2+^		2.24 × 10^−8^	3.63 × 10^−7^ – 1.06 × 10^−5^			WL: 10		
PPy/GOx^a^	Cu^2+^	Potentiometry	7.9 × 10^−8^	7.9 × 10^−8^ – 1.6 × 10^−5^	N.R.	Water and PBS 10–15′	SL: 8	98–101%^4^	Ayenimo and Adeloju, 2015
	Hg^2+^		2.5 × 10^−8^	2.5 × 10^−8^ – 5 × 10^−6^					
	Pb^2+^		2.4 × 10^−8^	1 × 10^−7^ – 1.5 × 10^−5^					
	Cd^2+^		4.4 × 10^−8^	4 × 10^−8^ – 6.2 × 10^−5^					
PANI-co-PDTDA/HRP^a^	Cd^2+^	DPV	7.11 × 10^−12^	0 – 8.89 × 10^−11^	Fe^2+^, Ni^2+^, Co^2+^, Na^+^, ${\text{SO}}_{4}^{2-}$, ${\text{PO}}_{4}^{3-}$	N.R.	SL: 5–7	96–104%^4^ 112–128%^5^	Silwana et al., 2014
	Pb^2+^		4.52 × 10^−12^	0 – 4.82 × 10^−9^					
	Hg^2+^		3.93 × 10^−12^	0 – 4.98 × 10^−9^					
N-CNTs/GOx^b^	Ag^+^	Amperometry	1.8 × 10^−9^	2 × 10^−8^ – 2 × 10^−7^	**Cu^2+^**; Co^2+^	PBS	WL: 6	N.R.	Rust et al., 2015
BSA/glycerol/β-galactosidase^d^	Cd^2+^	EIS	6.18 × 10^−8^	2.09 × 10^−8^ – 2.09 × 10^−1^	N.R.	N.R.	N.R.	95-103%^5^	Fourou et al., 2016
	Cr^6+^	EIS	1.76 × 10^−9^	5.65 × 10^−10^ – 5.65 × 10^−4^					
	Cd^2+^	SWV	6.76 × 10^−11^	2.09 × 10^−11^ – 2.61 × 10^−1^					
	Cr^6+^	SWV	1.76 × 10^−9^	5.65 × 10^−10^ – 5.65 × 10^−4^					
PNR/HRP^e^	Cr^3+^	Amperometry	0.27 μM	0.2 – 5.1 μM	Zn^2+^, Cu^2+^, Cd^2+^, Co^2+^, Ni^2+^, Hg^2+^, Pb^2+^	Acetate Buffer	SL: 21	N.R.	Attar et al., 2014
	Cr^6+^		1.6 μM	0.05 – 035 μM					

a *Platinum electrode (PtE)*.

b *Glassy carbon electrode rotating disk (GC-RDE)*.

c *Glassy carbon electrode (GCE)*.

d *Gold electrode (AuE)*.

e *Carbon film electrode (CFE)*.

1 *Some values have been transformed in molar concentration*.

2 *Interferents species are in bold character*.

3 *SL, Storage Life: days; WL, Working Life: number of consecutive measurements*.

*BSA, Bovine serum albumin; DPV, Differential pulse voltammetry; EIS, Electrochemical impedance spectroscopy; GOx, Glucose oxidase; HRP, Horseradish peroxidase; MT-MWCNTs, Maize tassel-multi-walled carbon nanotubes; N-CNTs, Nitrogen-doped carbon nanotubes; PANI-co-PDTDA, Poly(aniline-co-2,2-dithiodianiline); PNR, Poly neutral red; PPy, Poly pyrrole; SWV, Square wave voltammetry*.

*Range of percentage obtained by the analysis in real samples (^4^tap water, ^5^river water)*.

Passing to consider some peculiar aspects, Ag^+^ ions were successfully determined using an amperometric glucose biosensor based on immobilization of glucose oxidase on N-doped carbon nanotubes (N-CNTs) modified glassy carbon rotating disk electrode (GC-RDE) (Rust et al., 2015). The application of CNTs offers a large surface area to electrochemical process.

A copolymer of aniline and dithiodianiline (DTDA) for the immobilization of HRP is applied by Silwana et al. (2014) in an inhibition biosensor for Cd^2+^, Pb^2+^, and Hg^2+^. DTDA acts as a mediator for HRP. Accuracy in analysis of spiked real samples is reported, without consideration of the simultaneous presence of the heavy metals.

Moyo et al. (2014) describes a step toward green chemistry, as maize tassel, a renewable, natural, and non-edible source of polymeric material is employed as support for enzyme immobilization. In this respect, the maize tassels have some important desirable characteristics such as being mesoporous, high adsorption capacity, and the presence of functional active groups such as –OH, –COOH, –NH_2_, –C = O.

In another work (Fourou et al., 2016), biosensors based on β-galactosidase inhibition was prepared to detect Cd(II) and Cr(VI). Conductometric, EIS and SWV transduction were employed and high accuracy was achieved in determination of Cr(VI)-spiked river water.

The work of Ayenimo and Adeloju (2015) reported a new ultra-thin layer of polypyrrole with GOx immobilized for potentiometric detection of Cu^2+^, Hg^2+^, Cd^2+^, and Pb^2+^. The detection limits of Cu^2+^, Hg^2+^, Cu^2+^, and Pb^2+^ were lower than the conventional biosensor that using thicker layer of polymeric material. The present biosensor gets a rapid recovery of his activity when it is washed with water and stored in buffer phosphate for 5 min.

Speciation ability of Cr is reported in Attar et al. (2014). Even if the biosensor is sensitive to both Cr(III) and Cr(VI), authors claim, probably on the basis of the lower sensitivity for Cr(III) (I_50_ = 37 μM) in comparison to Cr(VI) (I_50_ = 3.8 μM), selective determination of Cr (VI) and Cr(III) in mixtures. It should be performed by measuring Cr(VI), then Cr(III) through the determination of total Cr by oxidation of Cr(III) to Cr(VI).However, irreversible Hg(II) inhibition can hinder its application.

## Biomimetic sensors based on electrosynthesized MIPs

Selective electrochemical sensors for ionic species based on electrosynthesized polymers have been reported since early work on conducting polymers (see e.g., Dong et al., 1988). Generally, anionic species (Dong et al., 1988) were the targets, but cationic species (see e.g., O'Riordan and Wallace, 1986) have also been considered. In this last case, two main approaches (Rahman et al., 2003) have been employed: cation exchange by reduced (neutral) polymers containing large (low-mobility) dopant anions and cation complexation by polymers bearing (or entrapping) ligands. In these systems selectivity is guaranteed by chemical recognition event or by electrode potential control. Even so, selectivity is still a crucial issue.

Selected examples of heavy metal electrochemical sensors based on electrosynthesized MIPs (e-MIPs), not or partially covered by previous reviews are included in Table 2: their analytical figures of merit are reported there. Comparison with Table 1 shows the significant improvement in selectivity and in stability employing MIP artificial receptors in place of biological components. An important feature is represented by high recovery values in application to real samples. Few examples of sensors based on nanomaterials have been found. Peculiar features can be underlined by considering each work.

**Table 2:** A summary of analytical characteristics of ion imprinted electrosynthetized polymers (IIPs) for heavy metals determination.

**Sensor**	**Metal Ions**	**Methods**	**Detection limit(M)^1^**	**Linear range(M)^1^**	**Tested interference^2^**	**Stability^3^**	**Recovery in real samples**	**References**
MWCNTs/Poly Arginine^b^	Cd^2+^	DPSV	4.94 × 10^−9^	1.99 × 10^−8^ – 9.87 × 10^−7^	Ascorbic acid, glucose, Fe^2+^, Hg^2+^, Zn^2+^	SL: 45	N.R.	Roy et al., 2014
	Cu^2+^		1.32 × 10^−8^	5.97 × 10^−8^ – 2.95 × 10^−6^				
	Pb^2+^		2.36 × 10^−9^	9.75 × 10^−9^ – 2.97 × 10^−7^				
MPMBT-${\text{SiO}}_{2}^{\text{a}}$	Hg^2+^	SWASV	1 × 10^−10^	1 × 10^−9^ – 1.6 × 10^−7^	Pb^2+^, Cd^2+^, Zn^2+^, Cu^2+^, Ag^+^	SL: 40	93.1–108.7%^5^	Fu et al., 2011
PPy/EBB^a^	Cu^2+^	DPASV Potentiometry	2 × 10^−9^	3.2 × 10^−8^ –1.0 × 10^−4^	**Hg^2+^**, **Ag^+^**, Ni^2+^, Co^2+^, Cd^2+^, Cr^3+^, Mn^2+^, Fe^3+^, Pb^2+^, Zn^2+^	SL: 30	97.1–98.4%^5^	Zanganeh and Amini, 2008
			1.0 × 10^−8^	5 × 10^−8^ – 1.0 × 10^−2^				
PANI/SSA^a^	Ag^+^	DPASV Potentiometry	2 × 10^−11^	1 × 10^−10^ –1 × 10^−7^, 1 × 10^−8^ – 1 × 10^−4^	**Hg^2+^**, **Cu^2+^**, Na^+^, K^+^, Mg^2+^, Ba^2+^, Zn^2+^, Al^3+^, Pb^2+^, Co^2+^, Hg^2+^, Cr^3+^, Cd^2+^, Ni^2+^	SL: 50	92.2–96%^5^	Hashemi and Zanganeh, 2016
			1 × 10^−9^	1 × 10^−8^ – 1 × 10^−3^				
PMB/Gly^a^	Cu^2+^	DPV	4.24 × 10^−11^	5 × 10^−10^ – 3 × 10^−8^	K^+^, Na^+^, Ca^2+^, Mg^2+^, Fe^3+^, Al^3+^, Cr^3+^, Mn^2+^, Hg^2+^, Cd^2+^, Ni^2+^, Zn^2+^, Co^2+^, Pb^2+^	N.R.	98.1%^6^ 95.5%^7^ 101.4%^8^ 98.1– 105.6%^9^	Li et al., 2015
PPy/EBB^a^	Ag^+^	Potentiometry	6.3 × 10^−9^	1 × 10^−8^ – 1 × 10^−1^, 3 × 10^10^ – 1 × 10^−7^, 1 × 10^−8^ – 1 × 10^−4^	**Hg^2+^**, Cd^2+^, Cu^2+^, Cr^3+^, Co^2+^, Mn^2+^, Fe^2+^, Fe^3+^, Ni^2+^, Pb^2+^	SL: 60	98%^10^	Zanganeh and Amini, 2007
		DPASV						
PPy/ARS^a^	Ag^+^	Potentiometry	2.5 × 10^−8^	5 × 10^−8^ – 6.3 × 10^−3^	K^+^, Na^+^, **Hg^2+^**, ${\text{NH}}_{4}^{+}$, Cd^2+^, Pb^2+^, Ba^2+^, Zn^2+^, Ni^2+^, Mn^2+^, Cu^2+^, Al^3+^, Cr^3+^	N.R.	102%^4^ 103%^5^ 102.2%^11^	Rounaghi et al., 2015
		Voltammetry	4.6 × 10^−10^	9.2 × 10^−10^ – 2.8 × 10^−6^				
MR/PPy^a^	Cu^2+^	Potentiometry	5.0 × 10^−7^	3.9 × 10^−6^ – 5.0 × 10^−2^	**Pb^2+^**, **Hg^2+^**, **Ag^+^**, Ni^2+^, Co^2+^, Cd^2+^, Cr^3+^, Ba^2+^, Fe^3+^, Al^3+^, K^+^, Na^+^	SL: 5	N.R.	Mazloum-Ardakani et al., 2013
		ASV	6.5 × 10^−9^	1.0 × 10^−8^–1.0 × 10^−3^				

a *Glassy carbon electrode (GCE)*.

b *Gold electrode (AuE)*.

1 *Some values have been transformed in molar concentration*.

2 *Interferents species are in bold character*.

3 SL, Storage Life: days

*Range of percentage recoveries obtained by the analysis in real samples (^4^tap water, ^5^river water, ^6^running water, ^7^fruit juice, ^8^rain water, ^9^beer, ^10^water samples, ^11^waste water sample). ASV, Anodic stripping voltammetry; ARS, Alizarin Red S; DPASV, Differential pulse anodic stripping voltammetry; DPSV, Differential pulse stripping voltammetry; DPV, Differential pulse voltammetry; EBB, Eriochrome Blue-Black B; Gly, Glycine; MPMBT, Microporous poly(2-mercaptobenzothiazole); MR, Methyl red; MWCNTs, Multi-walled carbon nanotubes; PANI, Poly aniline; PMB, Poly Methylene Blue; PPy, Poly pyrrole; SSA, 5-sulfosalicylic acid; SWASV, Square wave anodic stripping voltammetry*.

In a study (Zanganeh and Amini, 2007) polypyrrole was electropolymerized in presence of a counter anion with low mobility (EBB). Imprinting of specific sites for Ag^+^ in polymer matrix was obtained by an unusual process, i.e., the application of successive potential steps to the polymer in presence of Ag^+^. These steps produce a doping/dedoping of silver ions due to the reduction and oxidation steps on the polymer backbone with EBB anion charge compensation by ingress of Ag^+^. The sensor can be employed either in potentiometric and voltammetric DPASV mode. In this last case, selectivity and detection limit are quite improved. A very similar work has been performed for Cu^2+^ by the same authors (Zanganeh and Amini, 2008). Also Mazloum-Ardakani et al. (2013) apply the same approach but the dopant of polypyrrole was methyl red. They cite Zanganeh and Amini's work but apparently overlooking the fact that it contains the same imprinting method. Finally, polypyrrole (dopant Alizarin Red S) has been used by the same preparation method for an Ag^+^ sensor (Rounaghi et al., 2015), but authors do not ascribe they work to MIP field.

Also polyaniline (PANI) has been used (Hashemi and Zanganeh, 2016) in a scheme involving an imprinting process after polymerization. During synthesis, PANI is doped with a chelating agent, 5-sulfosalicylic acid (SSA). Doping/undoping cycles in the presence of Ag^+^ induces recognition sites in the polymer matrix for the cation with sensitivity, selectivity and chemical reversibility of the prepared sensor. Both voltammetric and potentiometric transduction has been employed with the first one showing lower detection limit, but the second one exhibiting a linear range up to 10^−3^ M.

A peculiar case is represented by the work described by Li et al. (2015), in which imprinting process is conjugated with a signal amplification process involving HRP to obtain a Cu^2+^ sensor with low detection limit and high selectivity. In fact, a novel MIP made of poly-methylene blue (PMB) based on copper-glycine (Cu-Gly) as template molecule was studied and a competitive scheme is applied in the detection. After rebinding with template, MIP is incubated in HRP-Cu-Gly solution in order to replace the Cu-Gly complex. Electrochemistry of HRP is employed as the transduction process and the decrease of the current caused by replacing HRP-Cu-Gly complex with Cu2^+^-Gly in the sample solution (produced by Gly addition) represents the sensor signal. Limits of detection by this approach can be reduced by three order of magnitudes compared with other Cu^2+^ imprinted polymers sensors as well as selectivity.

Also nanostructures has been applied in this field and employed to produce a system able to simultaneous detection of different heavy metals. In a study (Roy et al., 2014), multi-template imprinted nanowires on the surface of multi-walled carbon nanotubes (MW-CNTs) covered by a layer of polyarginine were electrosynthesized. Polyarginine was grown by electropolymerization of arginine molecules preadsorbed by electrostatic interactions on MW-CNT surfaces derivatized by -COOH groups. Template ions (Pb^2+^, Cd^2+^, Cu^2+^) can be then selectively detected by a differential pulse stripping voltammetry. The sensor has been successfully applied to several food and blood samples to investigate heavy metal uptake by human body.

A voltammetric sensor based on novel nanosized Ag(I)-imprinted polymer is presented by Zhiani et al. (2016). In relation to this work, it seems quite surprising that nanosize particles were obtained by bulk polymerization and crushing in a mortar even apparently without sieving. On the other hand, the SEM pictures included in the paper show nanoparticles are quite aggregated.

Surface imprinting is the strategy pursued by Fu et al. (2011). In this work core-shell particles originating from SiO_2_ microparticles, whose surface is covered by a thin film of poly(2-mercaptobenzothiazole), were grown. The monomer was the complex Hg^2+−^2-mercaptobenzothiazole. After washing out Hg^2+^, SiO_2_ is dissolved by an HF attack leaving microporous MIP particles sensitive to Hg^2+.^

## Conclusions

Electrosynthesized MIPs for heavy metals are promising recognition elements of biomimetic electrochemical sensors for these toxic species. Selectivity, sensitivity, and chemical reversibility seem distinct features of these devices. The possibility of multi-template imprinting represents a way to be explored to obtain simultaneous detection of single species, while speciation capability appears not to be considered at a sufficient extent. Enzyme inhibition electrochemical biosensor are overcome by the above sensors apart the ability to give an integrated response directly related the global toxicity of the sample.

## Author contributions

CM has conceived, designed the work, written and revised the manuscript. SD has collaborated to design, to write and revise the manuscript. EM has collaborated to design, to write and revise the manuscript.

### Conflict of interest statement

The authors declare that the research was conducted in the absence of any commercial or financial relationships that could be construed as a potential conflict of interest.
